# Supplementary material for: Ultrafast fMRI reveals serial queuing of information processing during multitasking in the human brain
Source: Nat Commun. 2025 Mar 29;16:3057. doi: 10.1038/s41467-025-58228-0 (PMC11953464; doi:10.1038/s41467-025-58228-0)
Supplement: Supplementary file 1 — Supplementary Information [file 41467_2025_58228_MOESM1_ESM.pdf]

Supplementary materials for

Ultrafast fMRI reveals serial queuing of information processing during multitasking in the human brain

Qiuhai Yue[1,2,*], Allen T. Newton[3], René Marois[2,4,5,*]

[1] School of Psychology, Shenzhen University, Shenzhen, China
[2] Department of Psychology, Vanderbilt University, Nashville, TN
[3] Vanderbilt University Medical Center, Nashville, TN
[4] Vanderbilt Vision Research Center, Vanderbilt University, Nashville, TN
[5] Vanderbilt Brain Institute, Vanderbilt University, Nashville, TN

[*] Corresponding authors:
Q. Y. (qyue@szu.edu.cn)
R. M. (rene.marois@vanderbilt.edu)

This document includes:

Supplementary Figs. 1-14

Supplementary Tables 1-3

Supplementary References

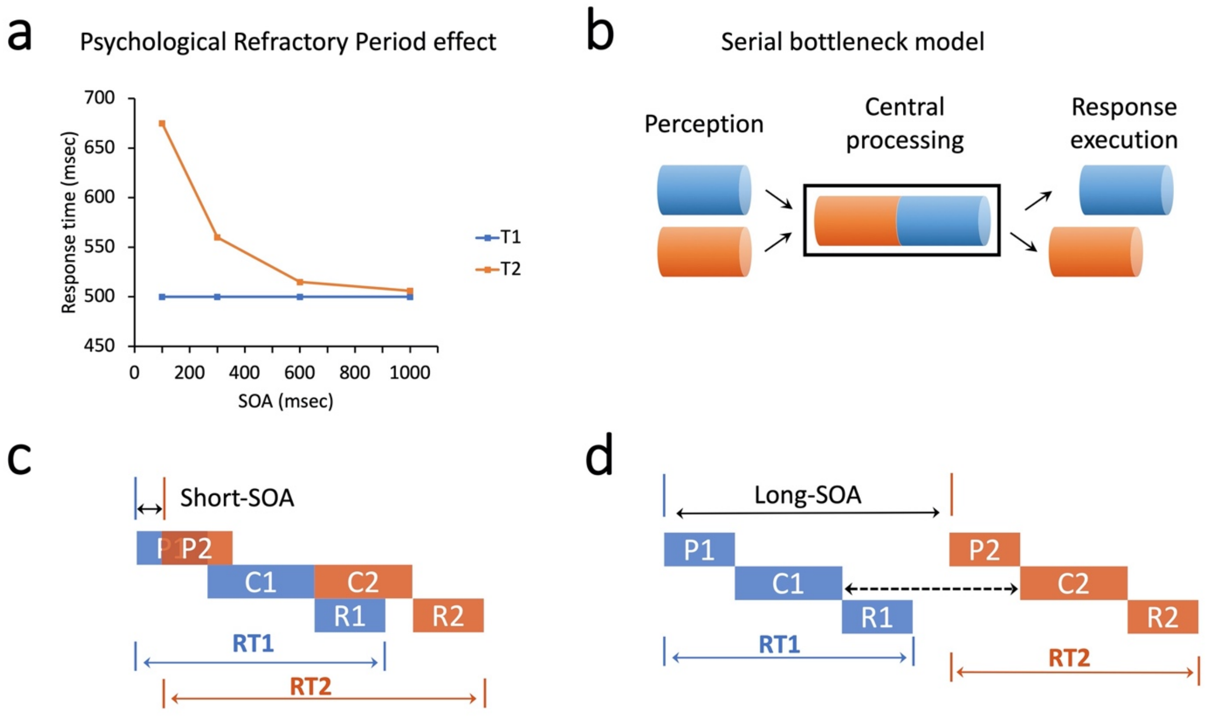

Supplementary Fig. 1. Psychological refractory period (PRP) and model of serial queuing of information processing in dual-task conditions. (a) A typical PRP effect shows progressively slowed RTs for the second (T2) of two distinct sensorimotor tasks as the stimulus-onset asynchrony (SOA) between the two tasks is decreased, whereas the RTs for the first task (T1) remains largely unaffected. **(b)** The serial bottleneck model posits that there is a central stage of information processing stage during which T1 and T2 can only be processed serially, while the two tasks can be processed in parallel at perceptual (P) and response (R) execution stages. **(c)** and **(d)** Model account of the PRP. At short SOA (c), central processing of Task 2 (C2) is delayed until central processing of Task 1 (C1) is completed, thus increasing RT2, whereas at long SOA (d) there is no delay as C1 is already completed by the time C2 is initiated, thus leading to shorter RT2.

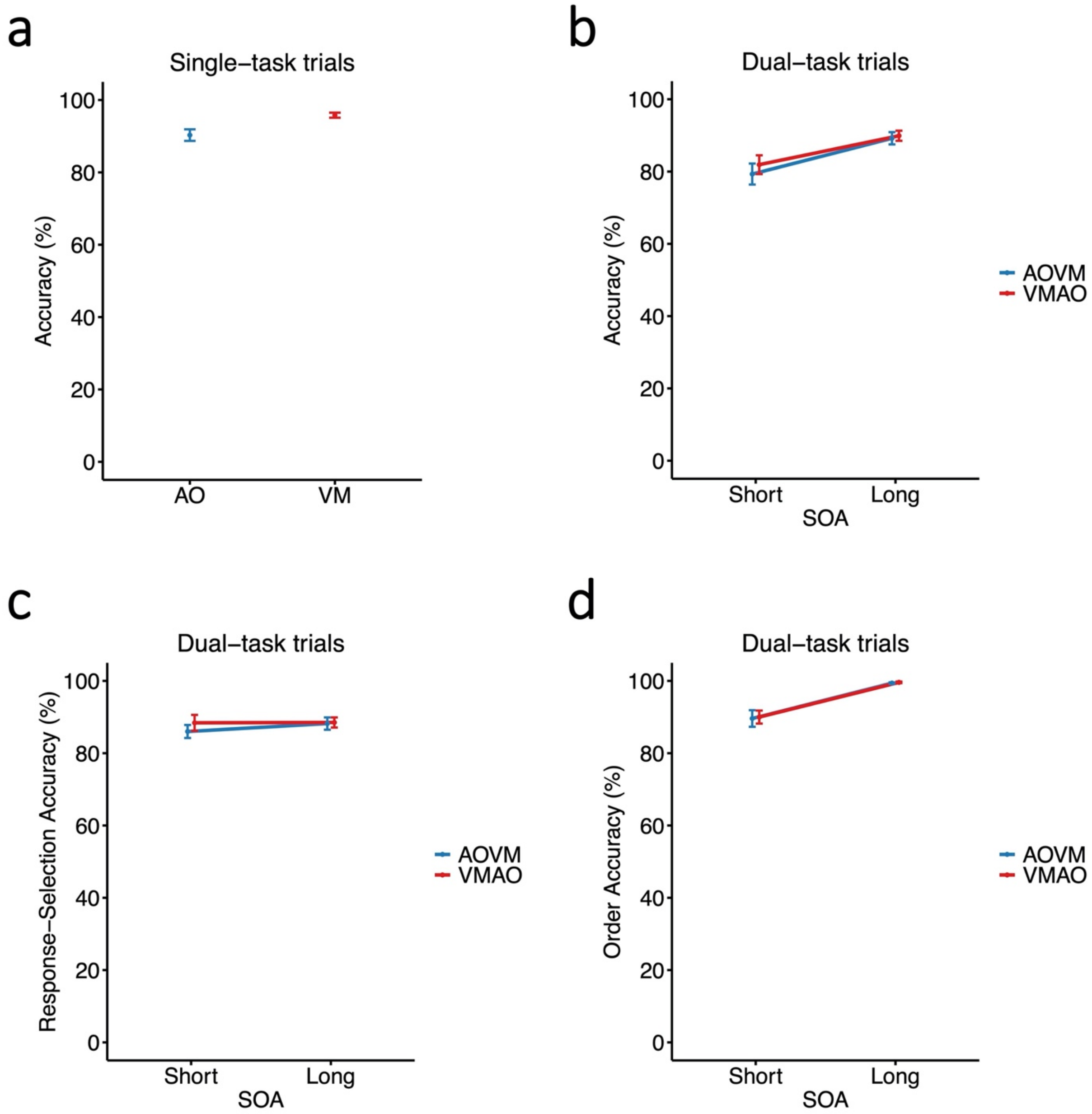

Supplementary Fig. 2. Accuracy performance. (a) In single-task trials, accuracy for the auditory-oculomotor (AO) task (90.3%) was lower than the visual-manual (VM) task (95.8%, t(25)=4.27, p=0.0002, Cohen's d=0.84, paired sample t test, two-tailed, N=26) which was probably due to a disrupt from scanning noise to the AO task. **(b)** Overall dual-task trials accuracy, defined as subjects responding to each task correctly and in the correct order. Overall accuracies for the short-SOA conditions (AOVM, 79.3%; VMAO, 81.9%) were lower than the long-SOA conditions (AOVM, 89.2%; VMAO, 89.9%) (F(1,25)=40.1, $p=1.26\times10^{-6}$, partial η^2=0.62, repeated measures ANOVA, N=26). There was neither a task order effect (F(1,25)=1.2, p=0.3, partial η^2=0.05), nor an SOA by task order interaction (F(1,25)=0.65, p=0.4, partial η^2=0.03); **(c)** Response selection accuracy (irrespective of the order of their response) was 86.0% for short-SOA AOVM, 88.4% for

short-SOA VMAO, 88.2% for long-SOA AOVM, and 88.5% for long-SOA VMAO. There were neither an SOA effect ($F(1,25)=2.34$, $p=0.14$, partial $\eta^2=0.09$) nor a task order effect ($F(1,25)=3.0$, $p=0.1$, partial $\eta^2=0.11$). The SOA by task order interaction was not significant ($F(1,25)=2.75$, $p=0.11$, partial $\eta^2=0.1$); **(d)** Response order accuracy – i.e. the accuracy in which participants responded in the same order as stimulus presentations irrespective of their accuracy in the specific response selection – was 89.6% for short-SOA AOVM, 90.0% for short-SOA VMAO, 99.4% for long-SOA AOVM, and 99.6% for long-SOA VMAO. There was a significant SOA effect ($F(1,25)=52.5$, $p=1.36\times10^{-7}$, partial $\eta^2=0.68$). There was neither a task order effect ($F(1,25)=0.34$, $p=0.9$, partial $\eta^2=0.013$), nor an SOA by task order interaction ($F(1,25)=0.01$, $p=0.98$, partial $\eta^2=0.0004$). Error bars represent the standard error of the mean. SOA, stimulus-onset asynchrony. Source data are provided as a Source Data file.

a

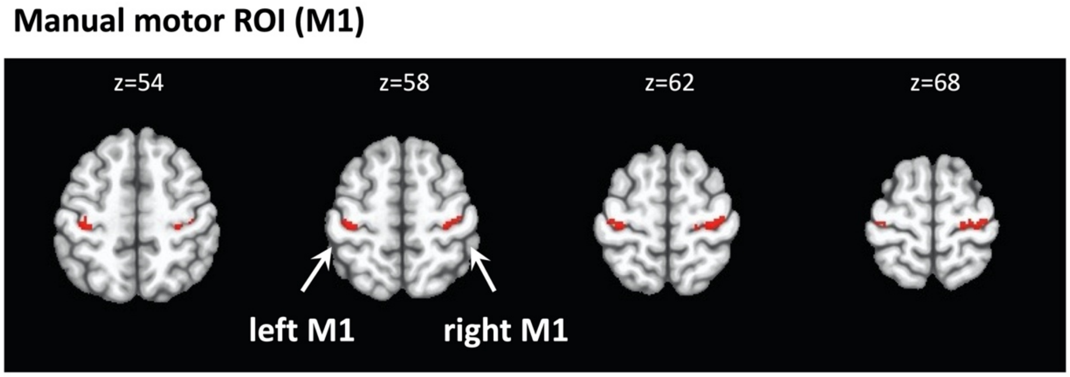

b

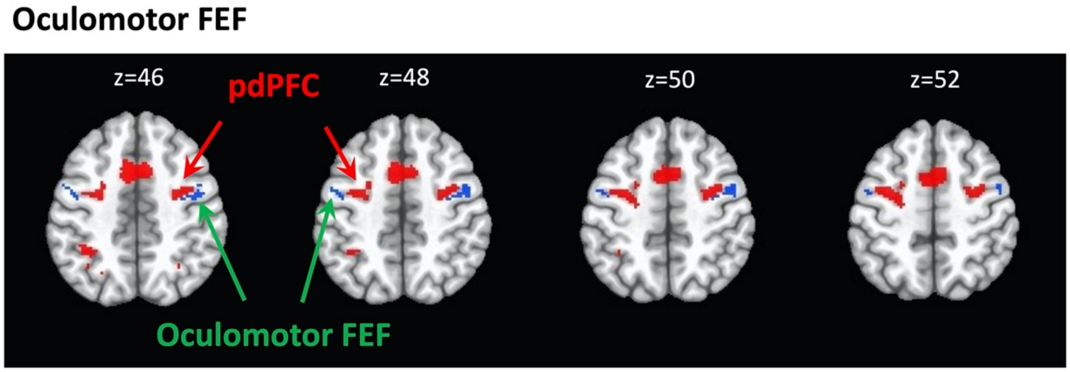

Supplementary Fig. 3. Motor regions definitions. (a) The manual motor ROI (M1, or primary motor cortex) was defined by intersecting the activated voxels to the manual motor response (voxel-level $p<0.005$, corrected at cluster level $\alpha<0.05$, with cluster size > 103 voxels) with an anatomical mask of the precentral gyrus. **(b)** The location of the oculomotor ROI (blue) corresponds to the canonical coordinates for the bilateral FEF (see Supplementary Table 1) and is more posterior and lateral to the pdPFC ROI (red) that is part of the MD network (see Supplementary Table 2). FEF, frontal eye field; pdPFC, posterior dorsal prefrontal cortex.

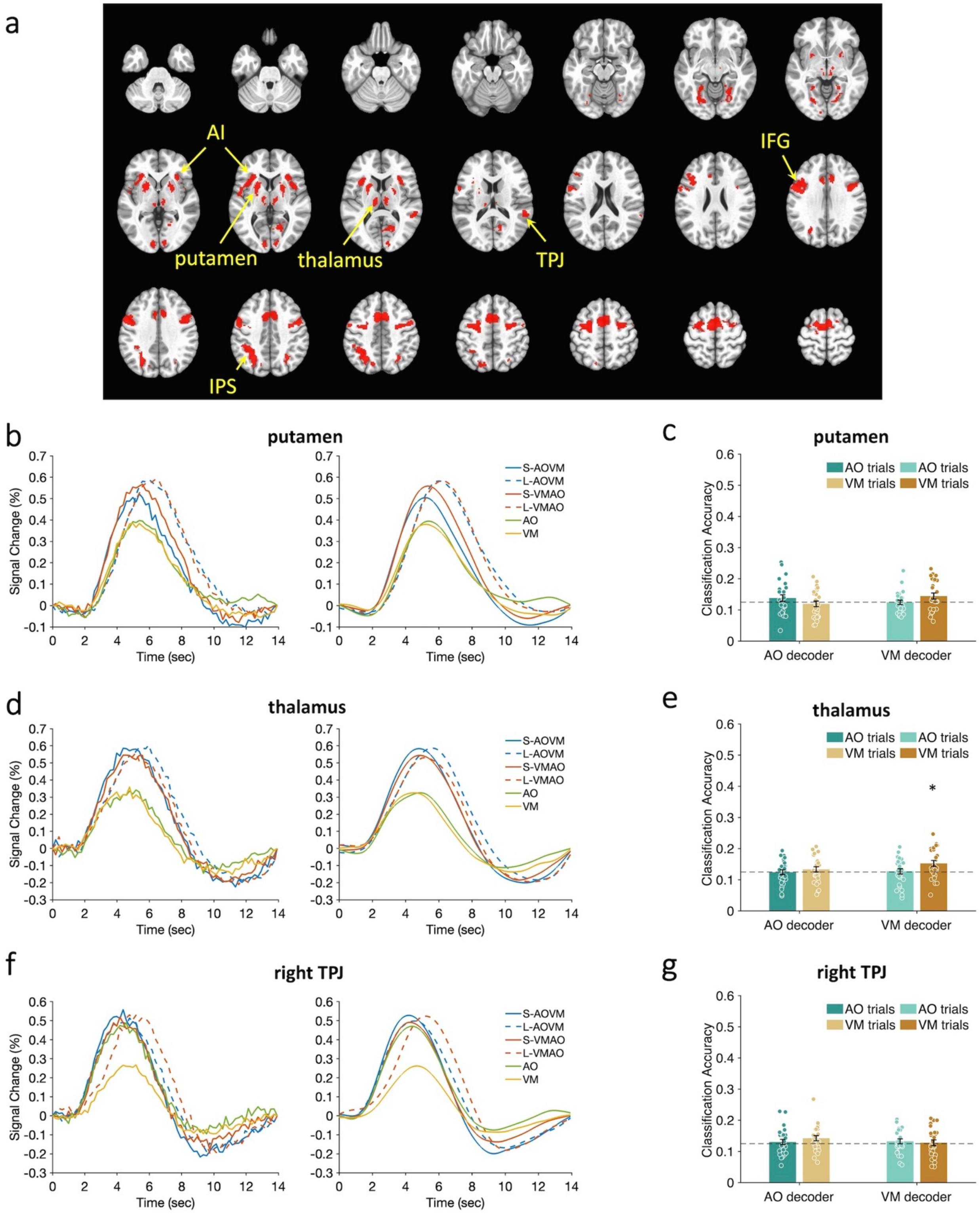

Supplementary Fig. 4. Univariate and multivariate analyses in the subcortical and the right TPJ ROIs isolated with the conjunction analysis. (a) Axial slices of SPMs showing overlapping activation for the auditory-oculomotor (AO) and visual-manual (VM) tasks in bilateral putamen,

bilateral thalamus and the right TPJ (see Supplemental Table 2). **(b, d)** Univariate blood-oxygen-level-dependent (BOLD) activation timecourses for the short-SOA AOVM (solid blue), long-SOA AOVM (dashed blue), short-SOA VMAO (solid red), long-SOA VMAO (dashed red), single AO (green) and single VM (yellow) trials in (b) bilateral putamen and (d) bilateral thalamus. The left columns show the raw group-averaged time courses of % signal change from the onset of T1, and the right column shows their fitted curves. Neither of the two subcortical areas exhibited postponed peak latencies in the dual-task trials relative to the single-task trials (putamen: 5.564sec for S-AOVM and 5.406sec for S-VMAO vs. 5.644sec for single-AO and 5.410sec for single-VM, p's>0.1, paired sample t test, two-tailed, N=26; thalamus: 4.892sec for S-AOVM and 4.867sec for S-VMAO vs. 5.027sec for single-AO and 4.796sec for single-VM, p's>0.5, paired sample t test, two-tailed, N=26). **(c, e)** Single-task multivariate decoding results in (c) bilateral putamen and (e) bilateral thalamus. The graphs show the group-averaged classification accuracy in the single task trials with color-filled dots representing individual data. The x-axis represents task-specific classifiers and colors represent different single-task trials. There was no significant task decoding except for the VM classifier applied to the VM trials in the thalamus (p=0.01, one sample t test, one-tailed, FDR corrected, N=26). Poor decoding in subcortical areas may at least be partly due to the small number of voxels contained in these small ROIs. This caveat notwithstanding, both the univariate and multivariate results provide little evidence for the notion that these subcortical regions are foci of the central bottleneck of information processing. **(f)** BOLD activations in single- and dual-task trials in the right TPJ ROI. The left columns show the raw results and the right columns show the fitted curves of the data. Although the right TPJ ROI showed robust activations in both the single-task and dual-task trials it did not exhibit significantly postponed peak latencies in the dual-task trials (4.534sec for S-AOVM and 4.697sec for S-VMAO) relative to the single-task trials (4.682sec for single-AO and 4.786sec for single-VM, p's>0.1, paired sample t test, two-tailed, N=26), contrary to the MD network ROIs. **(g)** Single-task multivariate decoding results. The TPJ failed to show any above-chance decoding in all single-task conditions (p's > .05, one sample t test, one-tailed, N=26). Taken together, these results are inconsistent with the TPJ playing a role in the central bottleneck of information processing. We postulate it may serve a more general function in behaviorally-relevant event detection[1]. TPJ, temporo-parietal junction; SOA, stimulus-onset asynchrony. Error bars represent the standard error of the mean. Asterisks indicate the significance of the decoding against chance level; * p<0.05. Source data are provided as a Source Data file.

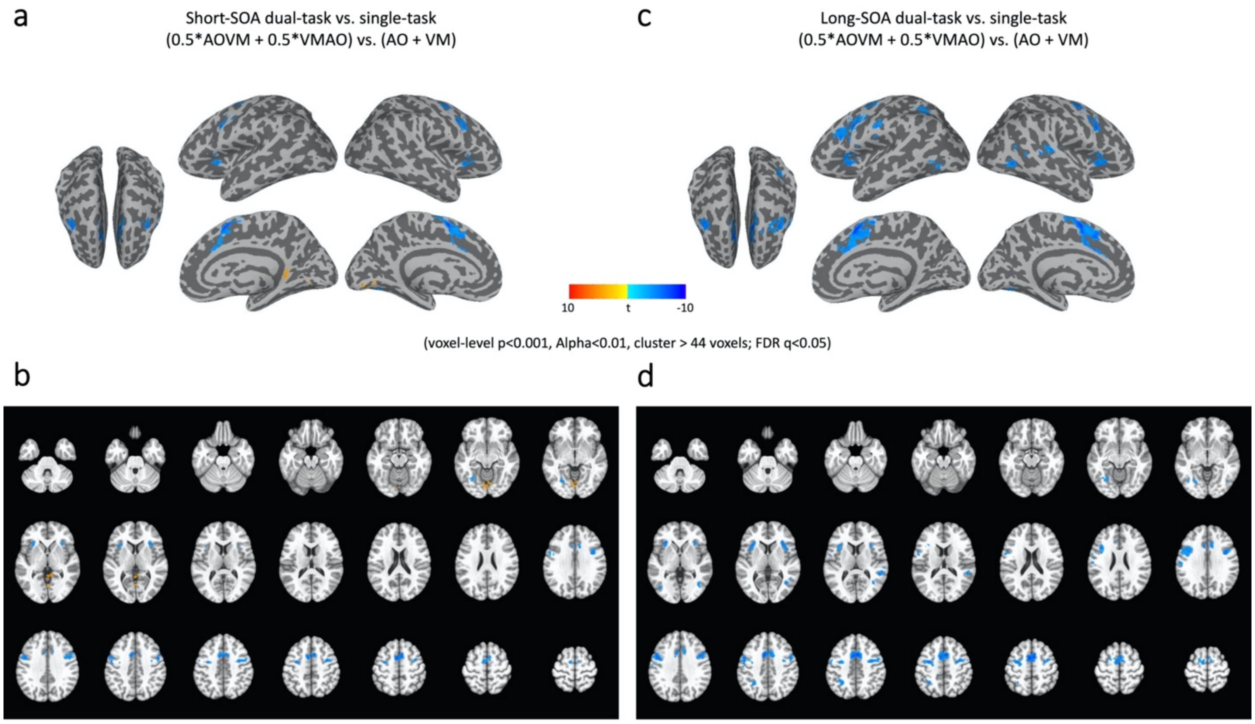

Supplementary Fig. 5. Dual-task vs. single-task SPMs. (a, b) Statistical parametric map (SPM) for the contrast of short-SOA dual-task trials vs. single-task trials shown on an inflated surface of a brain template (a) and axial slices (b). **(c, d)** SPM for the contrast of long-SOA dual-task trials vs. single-task trials shown on an inflated surface of a brain template (c) and axial slices (d). Results are reported based on a threshold at voxel level of $p<0.001$, and corrected at cluster level $\alpha<0.01$, with cluster size > 44 voxels. Warm-colored regions show greater activation for dual-task than single-task trials, and cold-colored regions show greater activation for single-task than dual-task trials. To account for the doubling of task presentations in dual-task conditions relative to single-task conditions, we balanced the contrasts by multiplying the dual-task weights by 0.5. This may explain the predominance of cold-colored foci in the present SPM, though none of these foci were in areas that weren't observed in the conjunction SPM. Note that the same contrast with unbalanced weights turned up more warm-colored areas but again in the same foci observed in the task-conjunction SPM. SOA, stimulus-onset asynchrony.

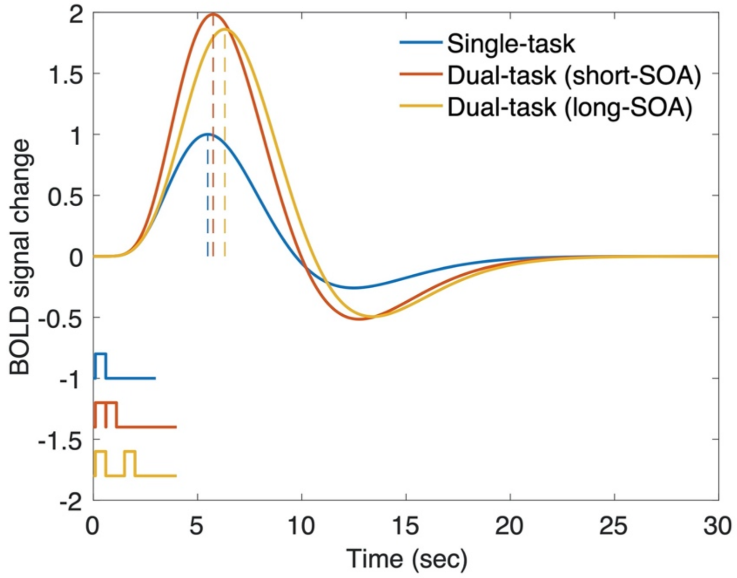

Supplementary Fig. 6. Simulation of blood-oxygen-level-dependent (BOLD) responses to single-task and dual-task trials. The graph shows simulated BOLD responses to a single-task trial (blue), a short-SOA dual-task trial (red), and a long-SOA dual-task trial (yellow). The response to the single-task trial is modeled by convolving the onset of a single neurophysiological event of 500ms-duration (i.e., the blue boxcar shown below the BOLD curves) with a classic double-gamma hemodynamic response function. The BOLD response timecourse to the short-SOA dual-task trial is modeled by two neurophysiological events of 500ms-duration separated by a 300ms SOA, with each event being modeled in the same way as in the single-task trial. The BOLD response timecourse to the long-SOA dual-task trial is modeled by two neurophysiological events of 500ms-duration separated by a 1500ms SOA. The three conditions not only differ in peak amplitudes but also in peak latencies (stippled vertical bars). Qualitatively similar results are obtained if the event duration is 1000ms instead of 500ms. SOA, stimulus-onset asynchrony; BOLD, blood-oxygen-level-dependent.

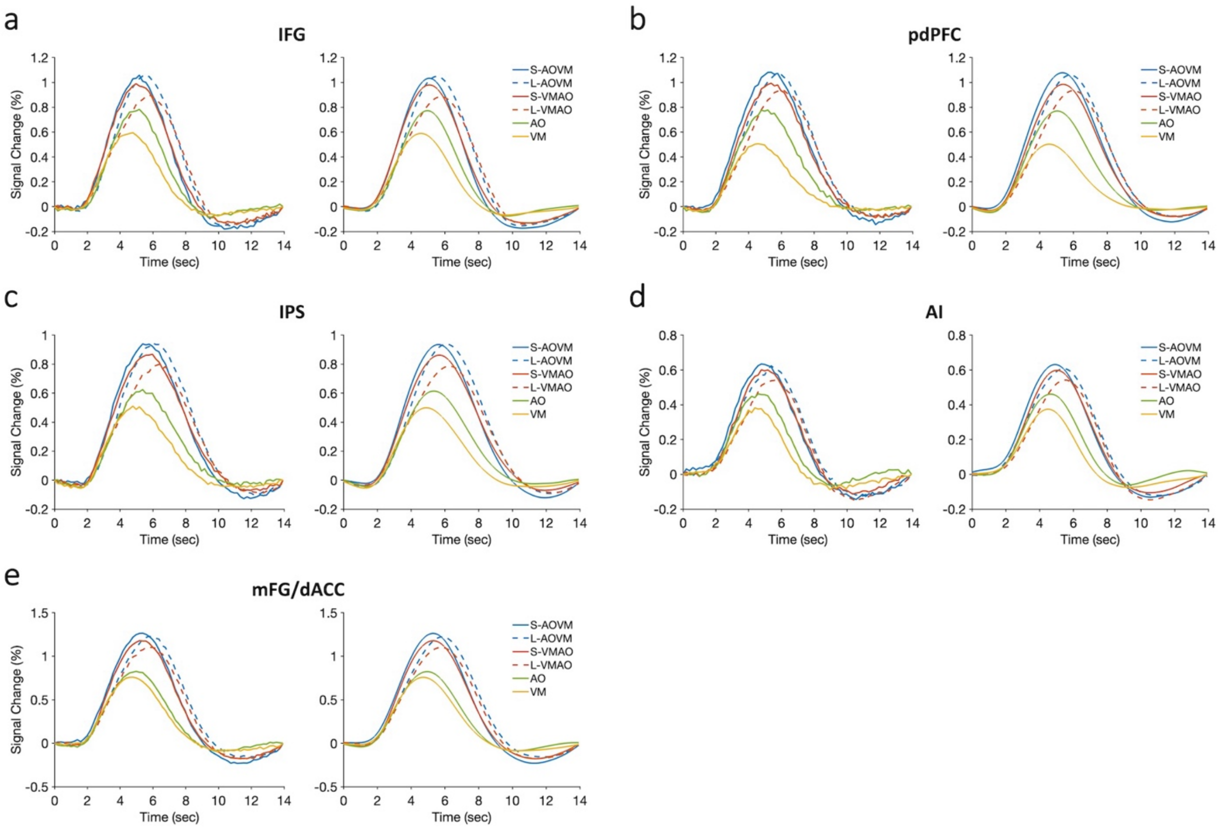

Supplementary Fig. 7. Univariate blood-oxygen-level-dependent (BOLD) activation timecourses in the single- and dual-task trials in the multiple-demand (MD) network ROIs. Group-averaged time courses of % signal change from the onset of T1 in short-SOA AOVM (solid blue), long-SOA AOVM (dashed blue), short-SOA VMAO (solid red), long-SOA VMAO (dashed red), single AO (green) and single VM (yellow) trials in **(a)** IFG, **(b)** pdPFC, **(c)** IPS, **(d)** AI, and **(e)** mFG/dACC. The left columns show the raw results and the right columns show the fitted curves of the data. IFG, inferior frontal gyri; pdPFC, posterior dorsal prefrontal cortex; IPS, intraparietal sulcus; AI, anterior insula; mFG/dACC, medial frontal gyri/dorsal anterior cingulate cortex; AO, auditory-oculomotor; VM, visual-manual; SOA, stimulus-onset asynchrony. Source data are provided as a Source Data file.

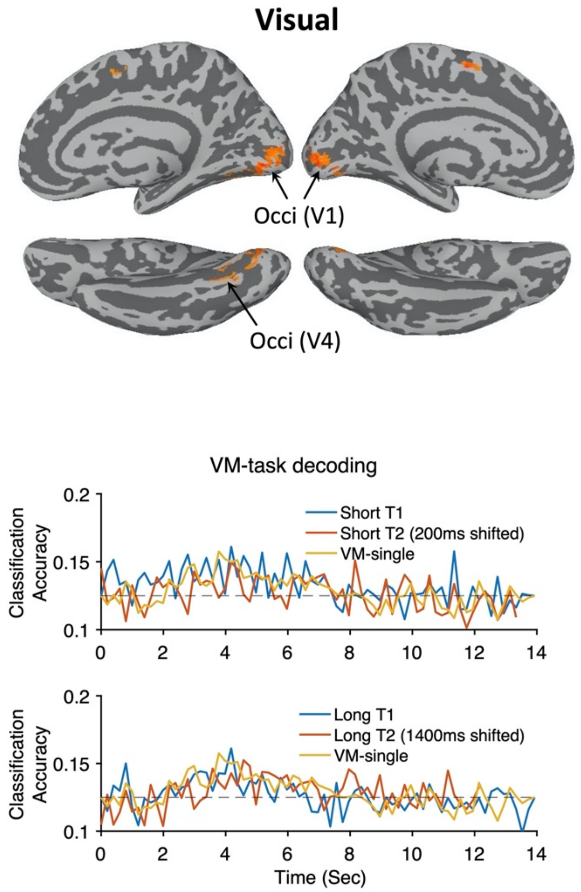

Supplementary Fig. 8. Visual-manual (VM) task decoding of dual-task trials in visual cortex ROIs. The upper graph shows SPMs for the contrast of visual vs. auditory inputs in single-task trials from a medial and an inferior view of an inflated surface of a brain template (see Fig. 3b). The lower graph shows the raw group-averaged classification accuracy time courses in the activated foci for the dual-task trials and single-task trials. Blue lines show VM task-specific decoding results for T1 in the dual-task conditions and red lines show VM task-specific decoding results for T2 in the dual-task conditions. Yellow lines show VM-specific decoding for the single VM task. Dashed lines represent chance level (12.5%). Source data are provided as a Source Data file.

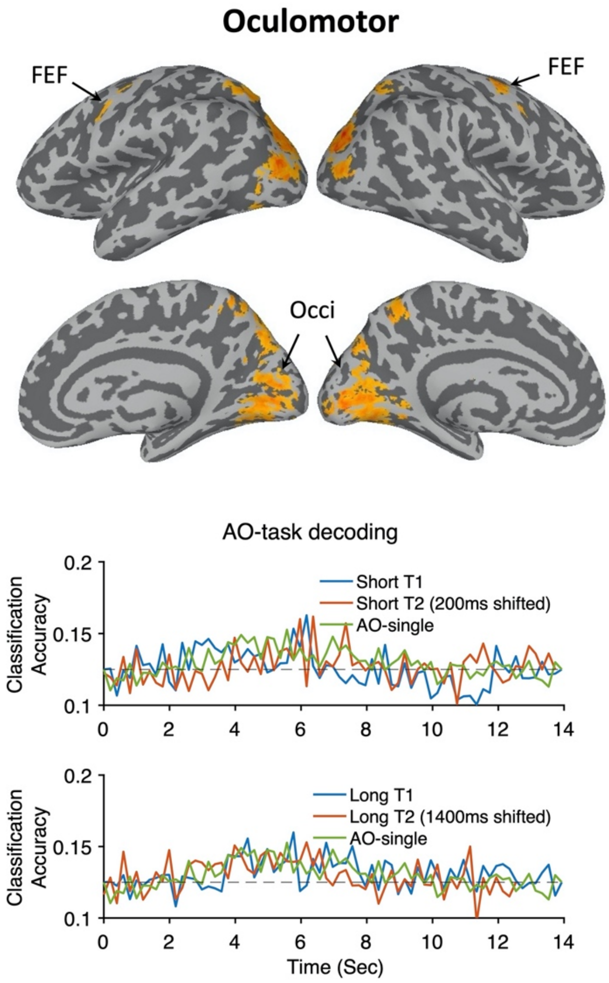

Supplementary Fig. 9. Auditory-oculomotor (AO) task decoding of dual-task trials in the oculomotor cortex ROI (FEF). The upper graph shows SPMs of the contrast of oculomotor vs. manual-motor responses in single-task trials (see Fig. 3c). The lower graph shows the raw group-averaged AO-specific decoding time courses in the dual-task trials and single-task trials in the bilateral FEF ROI. Blue lines show AO task-specific decoding results for T1 in the dual-task conditions and red lines show AO task-specific decoding results for T2 in the dual-task conditions. Green lines show AO-specific decoding for the single AO task. Dashed lines represent chance level (12.5%). FEF, frontal eye field. Source data are provided as a Source Data file.

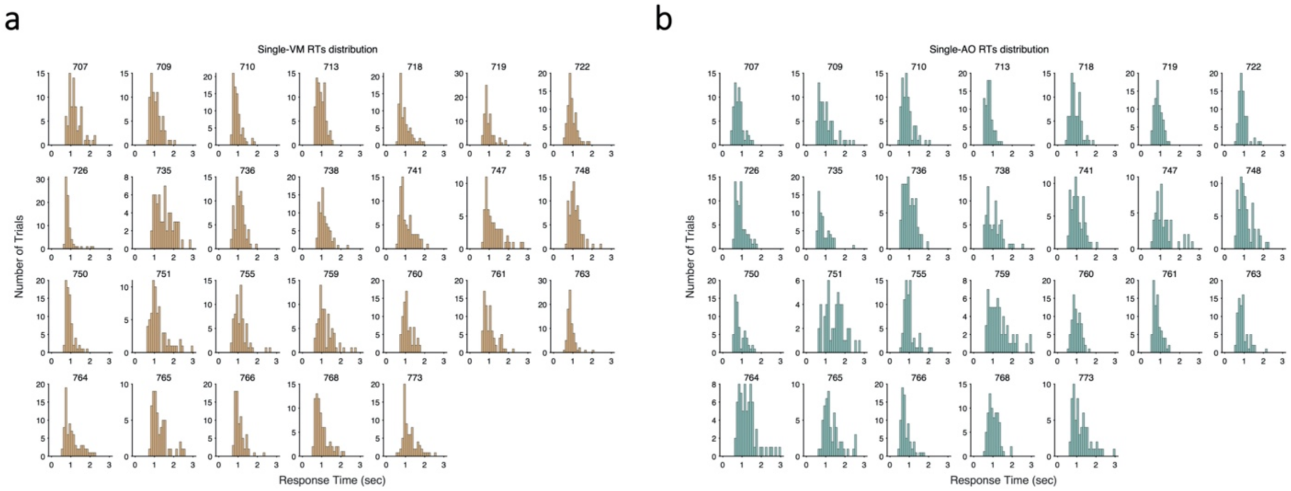

Supplementary Fig. 10. Individual participant RT variability in (a) single visual-manual (VM) and (b) single auditory-oculomotor (AO) tasks. The graphs show for each participant the histogram of trial RTs with a bin width of 0.1sec. Source data are provided as a Source Data file.

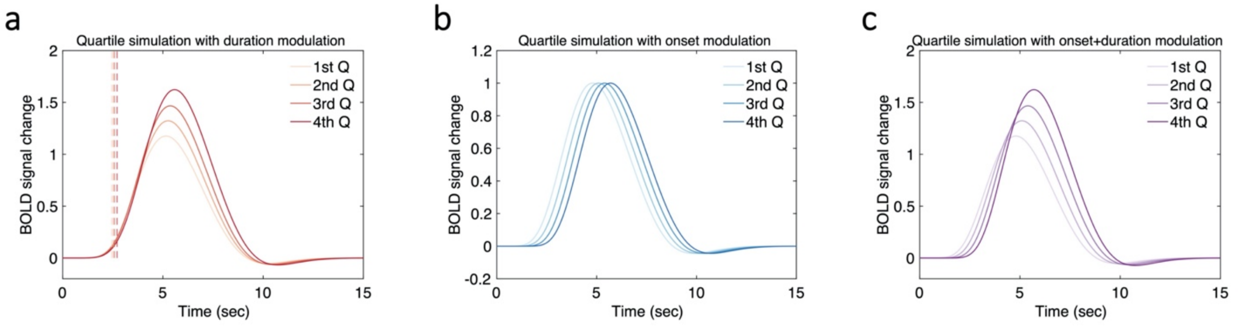

Supplementary Fig. 11. Simulation of the RT quartile effect on blood-oxygen-level-dependent (BOLD) responses to the single-task trials. (a) Quartile effect with differences in stimulus duration; **(b)** Quartile effect with differences in stimulus onset; **(c)** Quartile effect with differences in both stimulus onset and duration. Vertical dashed lines indicate small differences in BOLD response onset as a result of stimulus duration modulation. Q1, the first quartile; Q2, the second quartile; Q3, the third quartile; Q4, the fourth quartile.

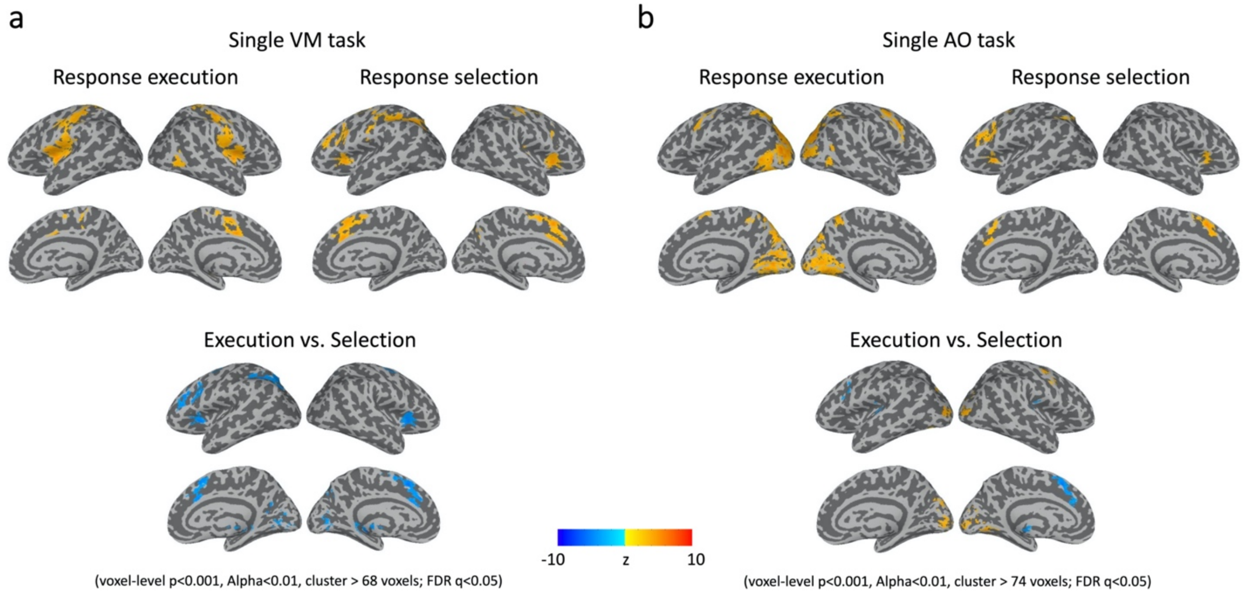

Supplementary Fig. 12. Comparisons of activated areas associated with response selection and response execution in the (a) single visual-manual (VM) task and (b) single auditory-oculomotor (AO) task. Response selection and response execution were teased apart based on the assumption that the duration of the response selection event is modulated by the response times (see main text) whereas response execution is a punctate event that takes place at the termination of response selection. Specifically, two regressors for each of the single tasks were included in the model (i.e., GLM1, see Methods): a response execution regressor that is modeled by convolving the motor response times (i.e., button press timing or saccade timing) with a fixed-duration (i.e., amplitude-unmodulated) double-gamma HRF, and a response selection regressor is modeled by convolving the motor response times with an amplitude-modulated double-gamma HRF in which the individual event for each trial is scaled with the response time for that trial. The upper panels show the SPMs for response execution and response selection vs baseline. The lower panels show the SPMs of the contrasts of response execution vs. response selection. The results of the single VM task are reported based on a threshold at voxel level of $p<0.001$ and corrected at cluster level $\alpha<0.01$, with cluster size > 68 voxels, and the results of the single-AO task are reported based on a threshold at voxel level of $p<0.001$ and corrected at cluster level $\alpha<0.01$, with cluster size > 74 voxels.

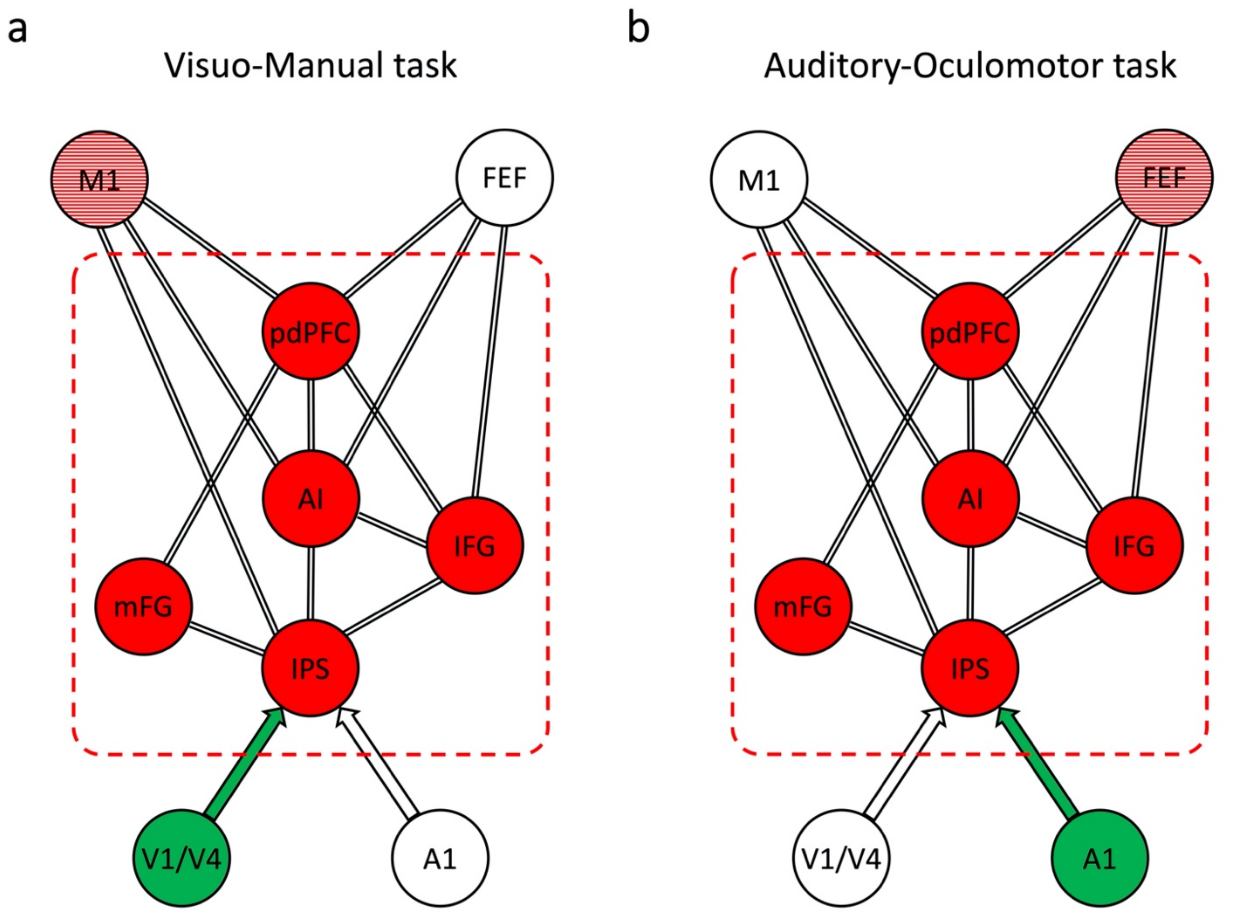

Supplementary Fig. 13. A flexible neural network model of the central bottleneck at the stage of response selection. The red dashed rectangle highlights the core multiple-demand (MD) network of IFG, pdPFC, IPS, AI, and mPF/dAC areas that form the central bottleneck of information processing in both **(a)** visual-manual (VM) and **(b)** auditory-oculomotor (AO) tasks. The MD network receives modality-specific sensory input depending on task context. Motor areas not only support response execution but also modality-specific response selection. The model proposes that response selection is achieved by the joint interaction between amodal (MD network) and modal (task-contingent motor) brain areas. Connections between brain areas within and outside the MD network are only for illustrative purposes. M1: motor cortex; V1/V4: visual cortex; A1: Auditory cortex; FEF, frontal eye field; IFG, inferior frontal gyri; pdPFC, posterior dorsal prefrontal cortex; IPS, intraparietal sulcus; AI, anterior insula; mFG/dACC, medial frontal gyri/dorsal anterior cingulate cortex.

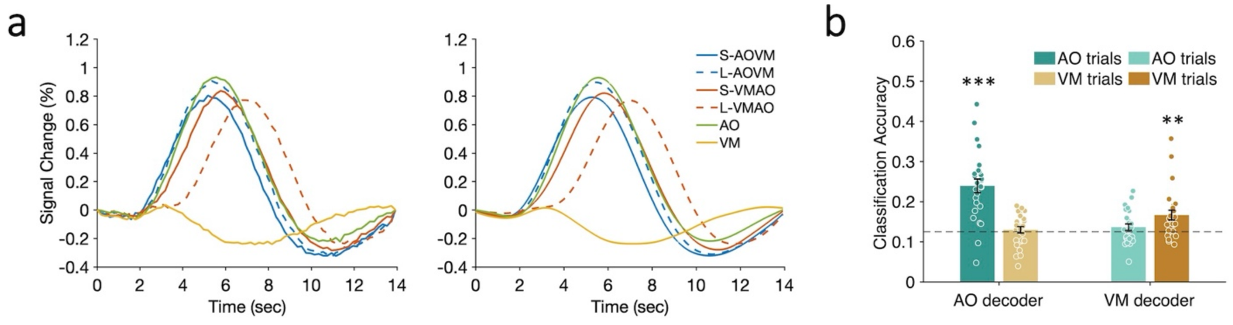

Supplementary Fig. 14. Univariate and multivariate analyses of single-task trials in the posterior parietal/superior occipital lobe. This ROI was isolated as an oculomotor area from the auditory-oculomotor (AO) vs. visual-manual (VM) contrast. Because this contrast gave a broad range of activations in the parietal and occipital areas, including the primary visual cortex in the medial and posterior occipital lobe (see Fig. 3 and Supplementary Table 1), a conservative threshold (voxel level of $p<0.0001$, and corrected at cluster level $\alpha<0.01$) was used to isolate a discrete cluster in the posterior parietal/superior occipital which differs from the primary sensory area. **(a)** The blood-oxygen-level-dependent (BOLD) timecourses are generally consistent with this ROI serving a role in oculomotor function: It responded robustly to all task conditions that included the AO task, but responded poorly or even was suppressed during the single VM task (e.g., significant deactivation relative to the baseline, $p=0.02$, paired-sample t test, two-tailed, N=26). **(b)** In contrast to the univariate results, the multivariate analyses revealed a pattern that is inconsistent with a purely oculomotor function for this brain region. As expected of an oculomotor area, single-task decoding results show that the classifier trained on AO task trials could distinguish between AO trials ($p<0.001$, one-sample t test, one-tailed, FDR corrected, N=26), but not between VM trials ($p=0.27$, FDR corrected). Unexpectedly, however, the VM classifier trained on the VM tasks could distinguish between VM task trials ($p=0.002$, FDR corrected), but not between AO task trials ($p=0.11$, FDR corrected), suggesting that it may encode visual sensory information. The incongruent findings between the univariate and multivariate analyses precludes us from making unequivocal claims about the role of this brain region in sensory, central or motor processing. Given that it exhibits both oculomotor activity and visual sensory information processing, we postulate that it may serve an integrated sensori-motor function in the visual modality[2]. Error bars represent the standard error of the mean. Asterisks indicate the significance of the decoding against chance level; ** $p<0.01$, ***$p<0.001$. Source data are provided as a Source Data file.

Supplementary Table 1. Activation foci for modality-specific processing

Regions	Hemi.	Coordinates (MNI)			Peak t value	Cluster size (voxel)
		x	y	z		
Auditory vs. visual						
HG/STG	left	-41	-21	5	11.07	1574
	right	53	-5	-1	10.90	1711
Visual vs. auditory						
Occipital (V1)	left	-11	-95	9	5.57	267
Occipital (V1-V4)	right	5	-81	-1	6.29	487
Medial	left	-3	-7	53	5.28	66
Oculomotor vs. manual-motor						
FEF	left	-46	-5	51	4.03	92
	right	45	-4	49	5.70	358
Occipito-temporo-parietal	left	-39	-81	9	9.37	5551
	right	29	-81	21	11.52	1867
Manual-motor vs. oculomotor						
Precentral/Postcentral Gyrus	left	-37	-33	51	8.26	725
	right	49	-25	47	9.07	628
Rolandic Operculum/Middle Insula	left	-43	-25	15	8.90	630
	right	41	-17	9	11.54	436
Medial	left	-1	-3	47	8.11	146
	right	3	-3	47	6.35	74
Cerebellum	right	9	-55	-13	8.44	175

Note: Auditory regions are defined by a contrast of auditory vs. visual inputs, masked by a contrast of auditory input vs. the fixation baseline. Visual regions are defined by a contrast of visual vs. auditory inputs, masked by a contrast of visual input vs. the fixation baseline. Oculomotor regions are defined by a contrast of oculomotor vs. manual-motor responses, masked by a contrast of oculomotor response vs. the fixation baseline. Manual-motor regions are defined by a contrast of manual-motor response vs. the fixation baseline, as well as a contrast of the left finger vs. the right finger responses. Results are reported based on a threshold at voxel level of $p<0.001$, and corrected at cluster level $\alpha<0.01$, with cluster size > 55 voxels. HG, Heschl's gyrus; STG, superior temporal gyrus; FEF, frontal eye field.

Supplementary Table 2. Activation foci from the conjunction analysis of auditory-oculomotor (AO) and visual-manual (VM) single tasks.

Regions	hemisphere	Coordinates (MNI)			Cluster size
		x	y	z	(voxel)
IFG	left	-49	8	34	378
	right	46	6	35	182
IPS	left	-31	-56	41	349
	right	29	-61	43	146
pdPFC	left	-29	-5	54	306
	right	32	-3	51	215
mFG	left	-6	6	52	522
	right	5	8	51	448
AI	left	-36	16	6	385
	right	35	19	6	295
Putamen	left	-22	7	4	327
	right	22	6	4	228
Thalamus	left	-10	-18	7	190
	right	9	-18	4	208
TPJ	right	58	-40	14	126
vOT	left	-25	-64	-9	237
	right	19	62	0	470
Occipital	left	-9	-91	2	102
	right	11	-89	4	123

Note: Results are reported based on a threshold at voxel level of $p<0.001$, and corrected at cluster level $\alpha<0.01$, with cluster size > 62 voxels. IFG, inferior frontal gyrus; IPS, intraparietal sulcus, pdPFC, posterior dorsal prefrontal cortex; mFG, medial frontal gyrus; AI, anterior insula; TPJ, temporo-parietal junction; vOT, ventral occipito-temporal. The vOT and occipital ROIs were most likely isolated with the conjunction SPM due to visual stimulus presentation in the VM task and to eye movements in the AO task.

Supplementary Table 3. Mean (Standard Deviation) of the hemodynamic response function (HRF) parameters for all six task conditions used in the subject-specific and condition-specific HRF modeling procedure

Parameter	AO	VM	Short-SOA AOVM	Short-SOA VMAO	Long-SOA AOVM	Long-SOA VMAO
*p*1	4.6 (0.5)	4.5 (0.7)	4.9 (0.5)	4.9 (0.4)	5.3 (0.5)	5.4 (0.4)
*f*1	3.9 (0.9)	3.8 (1.1)	4.4 (0.9)	4.5 (0.9)	4.7 (0.9)	5.2 (0.7)
*p*2	8.4 (2.8)	8.9 (1.9)	9.0 (1.6)	9.1 (1.4)	9.0 (1.1)	9.5 (1.3)
*f*2	3.5 (1.3)	4.0 (1.5)	4.1 (1.2)	4.2 (1.7)	4.6 (2.5)	4.6 (2.2)
dip	0.39 (0.23)	0.28 (0.32)	0.42 (0.27)	0.31 (0.21)	0.44 (0.23)	0.50 (0.22)

Note: *p*1 and *p*2 represent the time to peak, and *f*1, *f*2 represent the full width at half maximum of the two gamma functions, and *dip* represents the ratio that adjusts the amplitude of gamma2 (undershoot component) relative to gamma1 (positive component), in a double-gamma HRF as described in the main text. AO, auditory-oculomotor; VM, visual-manual; SOA, stimulus-onset asynchrony.

Supplementary References

1. Corbetta, M., Kincade, J. M., Ollinger, J. M., McAvoy, M. P., & Shulman, G. L. Voluntary orienting is dissociated from target detection in human posterior parietal cortex. *Nat. neurosci.* **3**, 292–297 (2000).
2. Kastner, S., & Ungerleider, L. G. Mechanisms of visual attention in the human cortex. *Annu. Rev. Neurosci.* **23**, 315–341 (2000).
