# Supplementary material for: Ultrafast fMRI reveals serial queuing of information processing during multitasking in the human brain
Source: Nat Commun. 2025 Mar 29;16:3057. doi: 10.1038/s41467-025-58228-0 (PMC11953464; doi:10.1038/s41467-025-58228-0)
Supplement: Supplementary file 2 — Reporting Summary [file 41467_2025_58228_MOESM2_ESM.pdf]

nature portfolio

Corresponding author(s): Qiuhai Yue, René Marois

Last updated by author(s): Feb 6, 2025

Reporting Summary

Nature Portfolio wishes to improve the reproducibility of the work that we publish. This form provides structure for consistency and transparency in reporting. For further information on Nature Portfolio policies, see our Editorial Policies and the Editorial Policy Checklist.

Statistics

For all statistical analyses, confirm that the following items are present in the figure legend, table legend, main text, or Methods section.

n/a	Confirmed	
☐	☒	The exact sample size (*n*) for each experimental group/condition, given as a discrete number and unit of measurement
☐	☒	A statement on whether measurements were taken from distinct samples or whether the same sample was measured repeatedly
☐	☒	The statistical test(s) used AND whether they are one- or two-sided<br>*Only common tests should be described solely by name; describe more complex techniques in the Methods section.*
☒	☐	A description of all covariates tested
☐	☒	A description of any assumptions or corrections, such as tests of normality and adjustment for multiple comparisons
☐	☒	A full description of the statistical parameters including central tendency (e.g. means) or other basic estimates (e.g. regression coefficient) AND variation (e.g. standard deviation) or associated estimates of uncertainty (e.g. confidence intervals)
☐	☒	For null hypothesis testing, the test statistic (e.g. *F*, *t*, *r*) with confidence intervals, effect sizes, degrees of freedom and *P* value noted<br>*Give P values as exact values whenever suitable.*
☒	☐	For Bayesian analysis, information on the choice of priors and Markov chain Monte Carlo settings
☒	☐	For hierarchical and complex designs, identification of the appropriate level for tests and full reporting of outcomes
☐	☒	Estimates of effect sizes (e.g. Cohen's *d*, Pearson's *r*), indicating how they were calculated

Our web collection on statistics for biologists contains articles on many of the points above.

Software and code

Policy information about availability of computer code

Data collection	Behavioral data was collected using Matlab (version: R2018b) with Psychtoolbox (version: 3.0.15) and ViewPoint (version: 2.9.5,142). fMRI data was obtained on a Philips 7-Tesla Achieva MRI scanner with a quadrature transmit and 32-channel receive head coil.
Data analysis	fMRI data was analyzed using AFNI (version: 7.14, Feb. 24, 2021), Freesurfer (version: 7.1.1), Matlab with the Princeton MVPA toolbox and the Multivariate Granger Causality (MVGC) toolbox (version: mvgc_v1.3).

For manuscripts utilizing custom algorithms or software that are central to the research but not yet described in published literature, software must be made available to editors and reviewers. We strongly encourage code deposition in a community repository (e.g. GitHub). See the Nature Portfolio guidelines for submitting code & software for further information.

Data

Policy information about availability of data

All manuscripts must include a data availability statement. This statement should provide the following information, where applicable:

- Accession codes, unique identifiers, or web links for publicly available datasets
- A description of any restrictions on data availability
- For clinical datasets or third party data, please ensure that the statement adheres to our policy

The raw neuroimaging data supporting the conclusions of this study is available at (https://s3.accre.vu:9000/maroislabbucket/maroisnatcomms/prp_raw_data.zip).

Research involving human participants, their data, or biological material

Policy information about studies with human participants or human data. See also policy information about sex, gender (identity/presentation), and sexual orientation and race, ethnicity and racism.

Reporting on sex and gender	Thirty-three adults (19-29 years old, mean: 23 years old; 6 males) participated in the fMRI study. After screening, the results reported in the manuscript are based on data from the remaining twenty-six subjects (19-29 years old, mean: 22 years old; 6 males). Sex and gender was determined based on self-reporting. Sex and gender was not considered in the present study, and findings apply to both sexes and genders, as no evidence indicate that the topics addressed in the present study apply only or differently to one sex/gender. In addition, the uneven sex/gender ratio of our data set would compromise the analysis and its power. Thus, no sex- and gender-based analyses were performed in the current study.
Reporting on race, ethnicity, or other socially relevant groupings	Socially relevant information was not considered in the present study, as no evidence indicate that the topics addressed in the present study apply only or differently to specific social groupings.
Population characteristics	See above
Recruitment	Participants were recruited from Vanderbilt University community via an online recruitment system at Department of Psychology at Vanderbilt University, and received monetary compensation for their participation in the current study.
Ethics oversight	The study procedure was approved by the Vanderbilt University Institutional Review Board, and Informed consent was obtained from all subjects.

Note that full information on the approval of the study protocol must also be provided in the manuscript.

Field-specific reporting

Please select the one below that is the best fit for your research. If you are not sure, read the appropriate sections before making your selection.

☒ Life sciences ☐ Behavioural & social sciences ☐ Ecological, evolutionary & environmental sciences

For a reference copy of the document with all sections, see nature.com/documents/nr-reporting-summary-flat.pdf

Life sciences study design

All studies must disclose on these points even when the disclosure is negative.

Sample size	Sample size was based on prior fMRI studies of dual-tasking in the Marois laboratory that revealed sufficient power with a target number of subjects of 25.
Data exclusions	Four subjects were discarded due to the technical issues with regard to either eye-tracking or button response recording. The data from three additional participants were not included in the analysis because their performances in at least one of the stimulus-response pairings were not different from chance, precluding the isolation of 'correct' trials.
Replication	All attempts at replication were successful. The univariate analyses that were similar to those used in prior studies replicated these earlier findings. We used leave-one-out cross-validation analyses for MVPA.
Randomization	We used a within-subject design for our principal analyses. We randomized Stimulus-Response pairings across participants, and trial presentation order and timing were randomized within participants.
Blinding	Not relevant to the present study.

Reporting for specific materials, systems and methods

We require information from authors about some types of materials, experimental systems and methods used in many studies. Here, indicate whether each material, system or method listed is relevant to your study. If you are not sure if a list item applies to your research, read the appropriate section before selecting a response.

Materials & experimental systems

n/a	Involved in the study
☒	☐ Antibodies
☒	☐ Eukaryotic cell lines
☒	☐ Palaeontology and archaeology
☒	☐ Animals and other organisms
☒	☐ Clinical data
☒	☐ Dual use research of concern
☒	☐ Plants

Methods

n/a	Involved in the study
☒	☐ ChIP-seq
☒	☐ Flow cytometry
☐	☒ MRI-based neuroimaging

Plants

Seed stocks: N.A.

Novel plant genotypes: N.A.

Authentication: N.A.

Magnetic resonance imaging

Experimental design

Design type: task state fMRI; event-related design

Design specifications: There were 80 trials per each of six experimental conditions (i.e., 4 dual-task conditions and 2 single-task conditions) across 10 runs; The experiment included both single-task and dual-task trials. In single-task trials, the visual or auditory stimulus was presented for 200ms, followed by a 2s window during which the participants' response could be recorded. In dual-task trials, both visual and auditory stimuli were presented with variable stimulus onset asynchrony (SOA) and task order. Specifically, the SOA was either short (300msec) or long (1500msec). The inter-trial intervals (ITI) (i.e., from T2 offset to the T1 onset of the next trial) were jittered between 3.98sec and 11.144sec, following a decay distribution with a mean of 5.4sec.

Behavioral performance measures: Two types of variables were measured. Manual responses (i.e., pressing buttons) and Oculomotor responses (i.e., eye movement) were recorded, and response times based on the correct responses were measured.

Acquisition

Imaging type(s): functional MRI

Field strength: 7-Tesla

Sequence & imaging parameters: Functional images were acquired by using a highly accelerated, 3D PRESTO EPI sequence to achieve both short TRs while maintaining BOLD-optimized TEs with short dynamic scan times. The parameters for functional scans were as follow: TR: 199ms, TE: 27.2ms, FA: 11 degree, matrix size: 80*80, in-plane voxel size: 2.7*2.7 mm2. Each scan had 1600 volumes (318.4sec), and for each volume there were 26 axial 4-mm thickness slices to cover the whole brain. An anatomical scan was also acquired for each subject using MPRAGE sequence with the following parameters: TR: 4.8ms; TE: 2.1ms, FA: 7 degree, TI: 1300, shot interval: 4500ms, 249 sagittal 0.7-mm thickness slices with in-slice matrix size: 352*352, voxel size: 0.7*0.7*0.7mm3.

Area of acquisition: Whole brain scan was used.

Diffusion MRI: ☐ Used ☒ Not used

Preprocessing

Preprocessing software: Neuroimaging data preprocessing was performed using AFNI (version: 7.14, Feb. 24, 2021) (Cox, 1996). First, individuals' anatomical images were skull-stripped using 3dQwarp via @SSwarper. Segmentation was then done on the anatomical image using Freesurfer (Fischl et al., 2004) to obtain masks for white matter, ventricles, and anatomical parcellation of grey matter for each subject. Standard preprocessing (using afni_proc.py) on functional images consisted of: head motion correction, functional-to-anatomical alignment using a local Pearson correlation algorithm (lpc+ZZ cost function in AFNI) (Saad et al.,

2009), whole-brain masking, and time-series local average scaling for interpreting effect estimates as percent signal change (Chen et al., 2017). No spatial smoothing was performed to preserve the spatial variances across neighboring voxels.

Normalization	All analyses were conducted in the subject's native space except for univariate group-level analyses which were performed in a standard space with the functional images being warped and registered to a template (MNI152_T1_2009c) and resampled at 2*2*2mm3.
Normalization template	MNI152_T1_2009c distributed in AFNI
Noise and artifact removal	Because multi-shot sequence in ultrafast scanning protocols is motion sensitive and vulnerable to physiological artifacts (cardiac and respiratory), some extra steps were performed to address these issues (Chen et al., 2019). Six head motion correction parameters and six temporal derivatives of head motion were also modeled as nuisance regressors in the general linear model. To address the physiological artifacts, six principal components extracted from white matter areas and six principal components extracted from ventricles were also modeled as nuisance in the general linear model to account for physiological noises (Behzadi et al., 2007; Muschelli et al., 2014).
Volume censoring	To reduce the influence of motion-induced artifacts, censoring was applied to the time points in which head motion exceeded a distance of 0.3mm (i.e., Euclidean norm) over the preceding time point or in which more than 10% of whole brain voxels were regarded as outliers by 3dToutcount (Taylor et al., 2018).

Statistical modeling & inference

Model type and settings	Mass univariate activation and multivariate pattern analysis (MVPA) were performed. At the first level, stimulus perceptual processes and motor responses, as well as whole-event for each of the experimental conditions were modeled, and at the second level, contrasts of auditory vs. visual stimuli, oculomotor vs. manual responses were performed.
Effect(s) tested	Contrasts of auditory vs. visual stimuli, oculomotor vs. manual responses were performed. Among the four dual-task conditions, a factorial design was used.

Specify type of analysis: ☐ Whole brain ☐ ROI-based ☒ Both

Anatomical location(s)	An anatomical mask (Glasser et al., 2016) was applied to the precentral gyrus to limit the activation foci to purely motor regions in an area corresponding to the primary motor cortex for manual responses
Statistic type for inference (See Eklund et al. 2016)	Cluster-wise. The activated areas are reported based on a threshold at voxel level of p<0.001, and corrected at cluster level a<0.01. Cluster size threshold was determined based on a Monte Carlo simulation approach (Cox et al., 2017).
Correction	We employed a Monte Carlo simulation approach to correct for multiple comparisons by using the -Clustsim option of the software tool 3dttest++ (AFNI), as it has been shown to effectively control the false positive rate under 5% (Cox et al., 2017).

Models & analysis

n/a	Involved in the study
☐	☒ Functional and/or effective connectivity
☒	☐ Graph analysis
☐	☒ Multivariate modeling or predictive analysis

Functional and/or effective connectivity	Granger causality (GC) analyses were conducted using the Matlab toolbox MVGC (Barnett & Seth, 2014). The order of the vector autoregressive (VAR) model was estimated using the Akaike information criterion (AIC). Then the VAR model was estimated for the selected model order, and pairwise-conditional Granger causalities was calculated in time-domain from VAR model parameters by state-space method (Barnett & Seth, 2015).
Multivariate modeling and predictive analysis	For MVPA, feature selection was conducted by choosing the 50 most activated voxels (i.e., top t values from individual subject GLM) to that sensory or motor condition relative to the baseline. A classifier was trained to discriminate among 8 alternative choices for each single task. We used the estimations (beta values) from GLM to train a task-specific classifier to discriminate a S-R mapping against the other 7 S-R mappings using L2-regularized logistic regression algorithm and set the penalty value at 25. The trained task-specific classifier was then applied to the corresponding task trials by using either a leave-one-out cross validation procedure, or a train-data1-test-data2 procedure. Classification accuracy was scored by counting the proportion of correctly classified trials. Significance was assessed at the group-level by comparing classification accuracy against a chance level (12.5%) by using a one-tailed (right) t test, and corrected for multiple comparisons in each ROI.
